# Sex differences in waist circumference obesity and eating speed: a cross-sectional study of Japanese people with normal body mass index

**DOI:** 10.3389/fnut.2024.1341240

**Published:** 2024-03-12

**Authors:** Yuri Yaguchi, Tsuneo Konta, Nahomi Imaeda, Chiho Goto, Yoshiyuki Ueno, Takamasa Kayama

**Affiliations:** ^1^Department of Education, Art, and Sciences, Yamagata University, Yamagata, Japan; ^2^Department of Public Health, Yamagata University Graduate School of Medical Science, Yamagata, Japan; ^3^Department of Nutrition, Shigakkan University, Obu, Japan; ^4^Department of Health and Nutrition, Nagoya Bunri University, Inazawa, Japan; ^5^Faculty of Medicine, Institute for Promotion of Medical Science Research, Yamagata University, Yamagata, Japan

**Keywords:** waist circumference obesity, normal weight, slow eating, diet, sex difference

## Abstract

**Background:**

Fast eating has been positively associated with visceral fat accumulation in normal-weight individuals according to body mass index (BMI). However, previous studies have not examined energy and nutrients, or adjusted for food intake. We examined the relationship between eating speed and visceral fat accumulation, using waist circumference as an index, in middle-aged participants who were considered to be of standard weight according to BMI, with nutrient intake added as an adjustment factor.

**Methods:**

We included 6,548 Japanese participants (3,875 men and 2,673 women) aged 40–74 years with BMI 18.5–25.0 kg/m^2^ who were enrolled in the Yamagata Cohort Study. Participants were divided into “fast,” “normal,” and “slow” eaters according to self-reported eating speed. Nutrient and food intake were evaluated using a food frequency questionnaire, and the difference in intake by eating speed and sex was compared. Logistic regression analysis was used to examine the relationship between waist circumference obesity (men ≥85 cm, women ≥90 cm, according to Japanese criteria) and eating speed, adjusted for nutrient intake and other lifestyle habits.

**Results:**

In men, slow eaters had greater intakes of dietary protein, fat, polyunsaturated fatty acids (PUFA), omega-3 PUFA, total dietary fiber, soluble dietary fiber, insoluble dietary fiber, soybean products, fish, green and yellow vegetables, other vegetables, mushrooms, and seaweed in comparison with normal-speed eaters. In men, waist circumference obesity was significantly lower among slow eaters than in the group with normal eating speed. In women, waist circumference obesity was not significantly associated with eating speed and was not also associated with nutrient/food consumption except omega-6 PUFA.

**Conclusion:**

Eating slowly was associated with healthy dietary habits. Our results could help prevent waist circumference obesity in men with a BMI between 18.5 and 25.0 kg/m^2^. However, similar findings were not observed in women, suggesting a sex difference.

## 1 Introduction

Abdominal obesity caused by visceral fat accumulation is a risk factor for metabolic and cardiovascular diseases and death from these diseases (1–3). Waist circumference (WC) has been used as a convenient indicator to estimate visceral fat accumulation. In Japan, WC shows a strong correlation with abdominal fat accumulation and risk factors of metabolic syndrome. Because WC is easy to measure, it is a commonly used screening tool for abdominal fat accumulation (4). Moreover, a Japanese case–control study showed that WC is associated with the risk of incident cardiovascular diseases (3).

“Normal-weight central obesity” has recently been defined as normal weight according to body mass index (BMI) but with abdominal fat accumulation. Normal-weight central obesity is a risk factor for metabolic and cardiovascular diseases, as well as related death (5–8). Therefore, prevention or improvement of visceral fat accumulation is important, regardless of BMI.

Cross-sectional and longitudinal studies have shown that fast eating is associated with higher body fat and BMI and with overweight or obesity (9–13). A systematic review and meta-analysis also showed that fast eating speed is positively associated with weight gain and BMI obesity risk (14). However, most previous studies have focused on obesity risk as identified using BMI. Regarding visceral fat accumulation, fast eating has recently been shown to be positively correlated with obesity risk in terms of visceral fat area size and WC (10, 15, 16); however, only one study has been conducted among people judged to have normal weight using BMI (15). Body fat accumulation is also related to the type and amount of energy, nutrients, and foods consumed. A previous study on the relationship between visceral fat accumulation and eating speed in normal-weight individuals considered the frequency of food group intake, but not the amount of energy, nutrients, or foods consumed (15).

The sex hormones estrogen and testosterone are involved in body fat distribution, with men having greater visceral fat accumulation (17). Lifestyle habits that lead to visceral fat accumulation, such as eating habits, physical activity, and smoking, also differ between men and women (5–8, 18). Therefore, to examine the relationship between visceral fat accumulation and eating speed, it is necessary to clarify the combined effect of several lifestyle habits for men and women.

Therefore, we examined the relationship between eating speed and WC-indexed visceral fat accumulation in men and women aged 40–74 years who were considered to have normal weight according to BMI, additionally adjusted for dietary habits and physical activity level (expressed as metabolic equivalents, or METs).

## 2 Materials and methods

### 2.1 Study population

The Yamagata Cohort Study was a community-based prospective cohort study that was part of a molecular epidemiological study in Yamagata Prefecture, Japan (19). The participants in the present study were individuals aged 40–74 years who underwent community-based annual health checkups and resided in one of seven cities (Yamagata, Sakata, Kaminoyama, Sagae, Higashine, Tendo, and Yonezawa) in Yamagata Prefecture, Japan. From 2009 through 2015, a total of 20,996 participants (8,568 men, 12,428 women) were enrolled. We excluded participants who did not provide information regarding their eating speed (*n* = 249), whose BMI was less than 18.5 or greater than 25 kg/m^2^ (*n* = 6,743), who did not complete the food frequency questionnaire (FFQ) (*n* = 7,281), and those whose total energy intake was extremely high (>4,000 kcal/day) or low (<1,000 kcal/day) or whose protein-derived energy ratio was more than 40% or whose fat-derived energy ratio was more than 60% (*n* = 175). Finally, 6,548 participants (3,875 men, 2,673 women) were included.

### 2.2 Anthropometric assessment

During annual health checkups, body weight, height, and WC were measured by trained nurses at each of the health checkup sites. BMI was calculated as participants’ body weight (kg) divided by the square of height (m). We defined WC obesity as abdominal fat accumulation, with a WC ≥85 cm for men and ≥ 90 cm for women, according to Japanese criteria (4).

### 2.3 Eating speed assessment

The Japanese health checkup questionnaire assesses eating speed using the question, “How fast is your speed of eating compared with other people?” Participants are asked to choose one of three responses for eating speed (slow, normal, and fast).

### 2.4 Dietary assessment

Dietary habits during the last year were assessed using the short version of a self-administered FFQ that has demonstrated validity and reproducibility (20–23). Daily consumption of staple foods (rice, bread, noodles including Japanese *soba*) was queried, with intake frequency divided into six categories (almost none, 1–3 times/month, 1–2 times/week, 3–4 times/week, 5–6 times/week, and every day). The amount of food consumed per meal at breakfast, lunch, and dinner was also queried (measured in bowls for rice/noodles and slices/rolls for bread). The intake frequency of alcoholic beverages (sake, Japanese *shōchū*, *shōchū* highball, large bottle of beer (633 mL), medium-sized bottle of beer (500 mL), 350 mL of canned beer, 250 mL of canned beer, single whiskey, double whiskey, and wine) was queried according to six categories (almost none, 1–3 times/month, 1–2 times/week, 3–4 times/week, 5–6 times/week, and every day) as well as the amount per day. For the other 43 food and beverage items, participants reported the frequency of food intake according to the following eight categories: almost never, 1–3 times per month, 1–2 times per week, 3–4 times per week, 5–6 times per week, once per day, twice per day, and ≥ 3 times per day. Intake of total energy and nutrients and 19 food groups were computed using a program developed by the Department of Public Health, Nagoya City University School of Medicine. A validation study was performed by comparing the intake of total energy and 26 nutrients estimated using the FFQ and 3-day weighed dietary records. Deattenuated, log-transformed, and energy-adjusted correlation coefficients for intake of total energy and 26 nutrients ranged from 0.10 to 0.86. The validity of deattenuated, log-transformed, and energy-adjusted correlation coefficients for energy-producing nutrients was 0.42 for protein, 0.51 for fat, and 0.74 for carbohydrates in men and 0.30 for protein, 0.40 for fat, and 0.57 for carbohydrates in women (21). Spearman’s within-class correlation coefficients for food group intakes for this short FFQ and the 3-day dietary records at 3-month intervals over four seasons (the gold standard for dietary surveys) were 0.51 for men and 0.47 for women, indicating that the validity estimates were good (23).

### 2.5 Evaluation of energy intake reporting

To identify any misreporting (under-reporting or over-reporting), we used the Goldberg and Black cutoff method (24). We calculated the ratio between reported energy intake (calculated from FFQ scores) and estimated basal metabolic rate obtained using the Ganpule formula (25). This ratio corresponded to physical activity level, as the participants in this study had a steady body weight. Daily physical activity was quantified using the Japan Public Health Center Physical Activity Questionnaire (26). Leisure time physical activity was quantified using the International Physical Activity Questionnaire (27) to quantify daily physical activity. The upper and lower cutoff of physical activity level for the 95% confidence limits were calculated for each participant. Those participants whose energy intake to basal metabolic rate ratio was below the lower physical activity level cutoff were considered under-reporters, whereas those whose energy intake to basal metabolic rate ratio was above the higher physical activity level cutoff were considered over-reporters.

### 2.6 Other assessment

To assess additional lifestyle habits, participants were asked about their breakfast and snack eating habits and their smoking habits. Women were also asked about their current menstrual status (menstruating, perimenopausal, or menopausal). Daily physical activity was calculated using the Japan Public Health Center Physical Activity Questionnaire and the International Physical Activity Questionnaire, and expressed in METs.

### 2.7 Statistical analysis

Using IBM SPSS ver. 29.0 (IBM Japan Inc., Tokyo, Japan), characteristics and lifestyle habits were compared between men and women and by eating speed for each sex. Before all statistical analyses, we adjusted nutrient intakes according to the energy density method. We tested the differences between eating speed and the prevalence of WC obesity and lifestyle habits (smoking, METs, breakfast intake, daytime and nighttime snack intake) and energy intake reporting using *χ*^2^ tests and residual analysis, and age, height, body weight, BMI, dietary energy, nutrient intake, and food group intake using t-tests or one-way analysis of variance after Tukey’s test.

To estimate the adjusted odds ratios (ORs) with 95% confidence intervals of WC obesity by eating speed, we used multivariable logistic regression with stratification by sex, adjusted for age, dietary nutrient consumption including alcohol intake, as well as smoking and METs as lifestyle habits. Probability values for statistical tests were two-tailed, and *p* < 0.05 or |Z| > 1.96 was regarded as statistically significant.

### 2.8 Ethical approval

This study was approved by the ethics committee of Yamagata University School of Medicine. The procedures were performed in accordance with the Declaration of Helsinki. All participants provided written informed consent prior to enrollment.

## 3 Results

Table 1 shows participant characteristics. Of participants, 25.7% of men and 4.3% of women had WC obesity; the prevalence of WC obesity was higher in men. Regarding eating speed, fewer women (6.5%) than men (8.1%) were slow eaters. A smaller proportion of women than men missed breakfast, but a higher proportion of women were sometimes eaters. Regarding the intake of daytime and nighttime snacks, 40.6% of women ate these every day compared with 14.2% of men. In contrast, the percentage of men who did not eat such snacks was 37.3% compared with 11.3% of women, a significant difference. Regarding under- and over-reporting of energy intake, significantly fewer women over-reported their energy intake. Physical activity levels (in METs) were significantly lower for women than for men. Significantly more men were smokers and significantly more women were nonsmokers.

**Table 1:** Characteristics of study participants according to sex.

	Men	Women	
	*n* = 3,875	*n* = 2,673	*p*-value^a^
Age, years	65.6 ± 7.4	59.9 ± 8.8^*^	
Height, cm	165.7 ± 6.2	155.0 ± 5.6^*^	
Body weight, kg	61.5 ± 6.2	52.2 ± 5.1^*^	
Body mass index, kg/m^2^	22.4 ± 1.6	21.7 ± 1.7^*^	
Waist circumference, cm	81.6 ± 5.2	79.3 ± 6.2^*^	
Men ≥85 cm, women ≥90 cm	995 (25.7%)	116 (4.3%)	<0.001
Men <85 cm, women <90 cm	2,880 (74.3%)	2,557 (95.7%)	
Eating speed			
Fast	863 (22.3%)	626 (23.4%)	0.042
Normal	2,698 (69.6%)	1,873 (70.1%)	
Slow	314 (8.1%)^b^	174 (6.5%)^b^	
Breakfast intake			
Every day	3,395 (87.6%)	2,320 (86.8%)	0.022
Sometimes	316 (8.2%)^b^	265 (9.9%)^b^	
Never	151 (3.9%)^b^	79 (3.0%)^b^	
Missing	13 (0.3%)	9 (0.3%)	
Daytime and nighttime snack intake			
Every day	549 (14.2%)^b^	1,084 (40.6%)^b^	<0.001
Sometimes	1,829 (47.2%)	1,262 (47.2%)	
Never	1,444 (37.3%)^b^	301 (11.3%)^b^	
Missing	53 (1.4%)	26 (1.0%)	
Misreporting of energy intake			
Under-reporting	1,391 (35.9%)	1,018 (38.1%)	0.008
Adequate reporting	2,421 (62.5%)	1,632 (61.1%)	
Over-reporting	63 (1.6%)^b^	23 (0.9%)^b^	
METs	40.1 ± 8.2	38.9 ± 7.2^*^	
Smoking habits			
Smoker	931 (24.0%)^b^	190 (7.1%)^b^	<0.001
Non-smoker	1,226 (31.6%)^b^	2,217 (82.9%)^b^	
Ex-smoker	1,717 (44.3%)^b^	230 (8.6%)^b^	
Missing	1 (0.0%)^b^	36 (1.3%)^b^	

Age, height, body weight, body mass index, waist circumference, and METS are shown as mean ± standard deviation. METs: metabolic equivalents.

^a^Chi-square test; ^b^|Z| > 1.96 (residual analysis); ^*^p < 0.05 (*t*-test).

Table 2 shows the characteristics of participants according to self-reported eating speed. In men, the fast-eating group had a significantly younger mean age and significantly greater height, weight, BMI, and WC than the group with normal eating speed. The group with slow eating speed had a significantly older mean age and significantly lower height, body weight, BMI, and WC than the group with normal eating speed. The proportion of men with a WC ≥85 cm was significantly lower among those with slow eating speed. In women, the fast-eating group had significantly higher body weight and BMI than the group with normal eating speed. The group with slow eating speed had significantly lower height and body weight than the group with normal eating speed. There were no differences in WC values and the proportion of women with a WC of ≥90 cm according to eating speed. In men, those who ate at a normal speed were lower percentage to not eat breakfast, while there was no such difference among women. Regarding snack intake, among men, a lower percentage of the fast eaters did not eat snacks, and a lower percentage of the slow eaters sometimes ate snacks. In contrast, among women, a higher percentage of the fast eaters ate snacks daily, whereas a higher percentage of the slow eaters did not eat snacks. Among men, fast eaters had significantly lower METs than normal eaters, but there was no significant difference in METs by eating speed for women. Misreporting of energy intake and smoking habits did not differ significantly according to eating speed for either sex.

**Table 2:** Characteristics of study participants according to self-reported eating speed.

	Men				Women			
Eating speed	Fast	Normal	Slow		Fast	Normal	Slow	
	*n* = 863	*n* = 2,698	*n* = 314	*p*-value^a^	*n* = 626	*n* = 1,873	*n* = 174	*p*-value^a^
Age, years	63.0 ± 8.1^*^	64.9 ± 7.3	66.3 ± 6.4^*^		59.5 ± 8.7	59.9 ± 8.8	60.9 ± 9.2	
Height, cm	166.7 ± 6.1^*^	165.5 ± 6.2	164.9 ± 6.0		155.5 ± 5.4	155.0 ± 5.5	153.6 ± 6.2^*^	
Body weight, kg	62.9 ± 6.2^*^	61.3 ± 6.1	59.6 ± 6.2^*^		52.9 ± 5.1^*^	52.1 ± 5.1	50.8 ± 4.9^*^	
Body mass index, kg/m^2^	22.6 ± 1.6^*^	22.4 ± 1.6	21.9 ± 1.7^*^		21.9 ± 1.7^*^	21.7 ± 1.7	21.5 ± 1.6	
Waist circumference, cm	82.2 ± 4.9^*^	81.6 ± 5.3	80.2 ± 5.5^*^		79.6 ± 6.3	79.2 ± 6.3	79.5 ± 5.9	
Men ≥85 cm, women ≥90 cm	243 (28.2%)	693 (25.7%)	59 (18.8%)^b^	0.005	29 (4.6%)	80 (4.3%)	7 (4.0%)	0.908
Men <85 cm, women <90 cm	620 (71.8%)	2,005 (74.3%)	255 (81.2%)^b^		597 (95.4%)	1,793 (95.7%)	167 (96.0%)	
Breakfast intake								
Every day	751 (87.0%)	2,372 (87.9%)	272 (86.6%)	0.042	523 (83.5%)	1,644 (87.8%)	153 (87.9%)	0.111
Sometimes	67 (7.8%)	227 (8.4%)	22 (7.0%)		77 (12.3%)	172 (9.2%)	16 (9.2%)	
Never	44 (5.1%)^b^	90 (3.3%)^b^	17 (5.4%)		25 (4.0%)	49 (2.6%)	5 (2.9%)	
Missing	1 (0.1%)	9 (0.3%)	3 (1.0%)^b^		1 (0.2%)	8 (0.4%)	0	
Daytime and nighttime snack intake								
Every day	136 (15.8%)	357 (13.2%)^b^	56 (17.8%)	0.009	298 (47.6%)^b^	722 (38.5%)^b^	64 (36.8%)	<0.001
Sometimes	427 (49.5%)	1,276 (47.3%)	126 (40.1%)^b^		267 (42.7%)^b^	913 (48.7%)^b^	82 (47.1%)	
Never	288 (33.4%)^b^	1,031 (38.2%)	125 (39.8%)		57 (9.1%)	216 (11.5%)	28 (16.1%)^b^	
Missing	12 (1.4%)	34 (1.3%)	7 (2.2%)		4 (0.6%)	22 (1.2%)	0	
Misreporting of energy intake								
Under-reporting	318 (36.8%)	976 (36.2%)	97 (30.9%)	0.360	256 (40.9%)	705 (37.6%)	57 (32.8%)	0.177
Adequate reporting	532 (61.6%)	1,679 (62.2%)	210 (66.9%)		364 (58.1%)	1,151 (61.5%)	117 (67.2%)	
Over-reporting	13 (1.55)	43 (1.6%)	7 (2.2%)		6 (1.0%)	17 (0.9%)	0	
METs	39.6 ± 7.9^*^	40.3 ± 8.3	39.2 ± 8.2		39.1 ± 7.2	39.0 ± 7.3	38.2 ± 6.4	
Smoking habit								
Smoker	202 (23.4%)	653 (24.2%)	76 (24.2%)	0.479	44 (7.0%)	133 (7.1%)	13 (7.5%)	0.428
Non-smoker	253 (29.3%)	878 (32.5%)	95 (30.3%)		513 (81.9%)	1,560 (83.3%)	144 (82.8%)	
Ex-smoker	408 (47.3%)	1,166 (43.2%)	143 (45.5%)		64 (10.2%)	150 (8.0%)	16 (9.2%)	
Missing	0	1 (0.04%)	0		5 (0.8%)	30 (1.6%)	1 (0.6%)	

Age, height, weight body mass index, and waist circumference are shown as mean ± standard deviation. METs: metabolic equivalents.

^a^Chi-square test; ^b^|Z| > 1.96 (residual analysis); ^*^p < 0.05 (Tukey’s honest significant difference test after one-way analysis of variance, comparison with normal group).

Regarding menstrual status, 92 (14.7%) in the fast-eating group, 269 (14.4%) in the normal-eating group, and 23 (13.3%) in the slow-eating group were menstruating; 489 (78.1%) in the fast-eating group, 1,453 (77.6%) in the normal-eating group, and 135 (77.6%) in the slow-eating group were menopausal; and 26 (4.2%) in the fast-eating group, 92 (4.4%) in the normal-eating group, and 6 (3.4%) in the slow-eating group were perimenopausal. Nineteen fast eaters, 59 normal-speed eaters, and 10 slow eaters did not respond (data not shown). Menstrual status was not significantly it associated with eating speed.

Table 3 shows the dietary energy, nutrient, and food group intake according to sex. Only energy, carbohydrate, and alcohol intake were higher in men than in women. The intake of other nutrients was higher in women than in men. Men consumed more rice and alcoholic beverages than women, but the consumption of other foods was higher in women.

**Table 3:** Intake of energy, nutrient, and food group volume, by eating speed in men and women.

	Men	Women	
	*n* = 3,875	*n* = 2,673	*p*-value*
Energy and nutrients			
Energy, kcal	1,920 ± 393	1,532 ± 264	<0.001
Protein, %energy	11.8 ± 2.1	13.5 ± 2.0	<0.001
Fat, %energy	19.2 ± 5.4	26.2 ± 6.0	<0.001
Saturated fatty acids, %energy	5.2 ± 1.3	6.8 ± 1.5	<0.001
Monounsaturated fatty acids, %energy	7.4 ± 2.1	10.1 ± 2.4	<0.001
PUFA, %energy	6.3 ± 1.9	8.4 ± 2.1	<0.001
Omega-3 PUFA, %energy	1.09 ± 0.35	1.40 ± 0.36	<0.001
Omega-6 PUFA, %energy	5.17 ± 1.63	6.93 ± 1.87	<0.001
Carbohydrates, %energy	61.3 ± 9.5	57.3 ± 8.4	<0.001
Alcohol, %energy	7.8 ± 7.2	3.0 ± 4.8	<0.001
Total dietary fiber, g/1,000 kcal	5.9 ± 1.9	8.1 ± 2.3	<0.001
Soluble dietary fiber, g/1,000 kcal	1.1 ± 0.4	1.5 ± 0.4	<0.001
Insoluble dietary fiber, g/1,000 kcal	4.0 ± 1.3	5.7 ± 1.6	<0.001
Food group (g/1,000 kcal)			
Rice	224.6 ± 68.7	176.3 ± 60.2	<0.001
Bread	13.1 ± 20.9	19.5 ± 20.8	<0.001
Noodles	37.2 ± 33.6	39.7 ± 29.8	0.002
Potatoes	7.3 ± 7.0	11.9 ± 9.3	<0.001
Confectionary	7.5 ± 8.9	12.5 ± 11.3	<0.001
Oils	6.7 ± 4.7	10.5 ± 6.0	<0.001
Soybean products	41.4 ± 23.9	49.6 ± 26.3	<0.001
Fish	29.7 ± 19.5	35.5 ± 18.5	<0.001
Meat	16.5 ± 11.1	24.3 ± 13.1	<0.001
Eggs	8.5 ± 8.4	11.7 ± 8.6	<0.001
Milk	55.5 ± 54.1	87.1 ± 69.0	<0.001
Green and yellow vegetables	33.5 ± 27.5	56.5 ± 37.7	<0.001
Other vegetables	29.5 ± 22.0	51.6 ± 31.7	<0.001
Fruits	22.2 ± 23.6	42.0 ± 38.0	<0.001
Mushrooms	3.1 ± 3.4	6.7 ± 5.8	<0.001
Seaweed	0.8 ± 0.9	1.2 ± 1.2	<0.001
Coffee	52.8 ± 54.4	95.2 ± 69.5	<0.001
Green tea	116.8 ± 118.8	167.9 ± 144.3	<0.001
Alcoholic beverages	129.2 ± 119.3	49.9 ± 79.3	<0.001

Data are shown as mean ± standard deviation.

^*^: *t*-test, comparison with all male and female participants.

PUFA, polyunsaturated fatty acids.

Table 4 shows the dietary energy and nutrient consumption of participants according to eating speed. There was no difference in energy intake according to eating speed between men and women. In men, fast eaters had greater intake of monounsaturated fatty acids than normal-speed eaters, and slow eaters had higher intakes of dietary protein, fat, polyunsaturated fatty acids (PUFA), omega-3 PUFA, total dietary fiber, soluble dietary fiber, and insoluble dietary fiber than normal-speed eaters. In women, fast eaters had lower intake of omega-6 PUFA than normal-speed eaters.

**Table 4:** Intake of energy and nutrient volume by eating speed in men and women.

	Men			Women		
Eating speed	Fast	Normal	Slow	Fast	Normal	Slow
	*n* = 863	*n* = 2,698	*n* = 314	*n* = 626	*n* = 1,873	*n* = 174
Energy, kcal	1,909 ± 396	1,924 ± 394	1,918 ± 372	1,535 ± 292	1,532 ± 258	1,512 ± 222
Protein, %energy	11.7 ± 2.0	11.8 ± 2.1	12.3 ± 2.4^*^	13.4 ± 2.2	13.5 ± 1.9	13.5 ± 2.4
Fat, %energy	19.3 ± 5.7	19.0 ± 5.3	19.8 ± 5.9^*^	26.0 ± 6.3	26.2 ± 5.9	27.0 ± 6.3
Saturated fatty acids, %energy	5.1 ± 1.3	5.2 ± 1.3	5.3 ± 1.4	6.8 ± 1.5	6.8 ± 1.4	7.0 ± 1.7
Monounsaturated fatty acids, %energy	7.6 ± 2.3^*^	7.4 ± 2.0	7.6 ± 2.4	10.1 ± 2.5	10.1 ± 2.4	10.3 ± 2.5
PUFA, %energy	6.4 ± 2.1	6.3 ± 1.8	6.6 ± 2.1^*^	8.3 ± 2.2	8.4 ± 2.1	8.6 ± 2.3
Omega-3 PUFA, %energy	1.09 ± 0.36	1.08 ± 0.34	1.15 ± 0.40^*^	1.39 ± 0.36	1.40 ± 0.35	1.43 ± 0.38
Omega-6 PUFA, %energy	5.26 ± 1.76	5.12 ± 1.57	5.33 ± 1.77	6.77 ± 1.88^*^	6.97 ± 1.86	7.04 ± 1.96
Carbohydrates, %energy	60.9 ± 9.8	61.5 ± 9.2	60.6 ± 10.2	57.3 ± 8.5	57.3 ± 8.3	56.4 ± 8.5
Alcohol, %energy	8.2 ± 7.5	7.7 ± 7.0	7.4 ± 7.4	3.3 ± 5.1	2.9 ± 4.6	3.0 ± 5.2
Total dietary fiber, g/1,000 kcal	5.9 ± 1.8	5.9 ± 1.9	6.3 ± 2.3^*^	8.0 ± 2.2	8.1 ± 2.3	8.3 ± 2.4
Soluble dietary fiber, g/1,000 kcal	1.1 ± 0.4	1.1 ± 0.4	1.2 ± 0.4^*^	1.5 ± 0.4	1.5 ± 0.4	1.5 ± 0.5
Insoluble dietary fiber, g/1,000 kcal	4.0 ± 1.3	4.0 ± 1.3	4.3 ± 1.6^*^	5.6 ± 1.6	5.7 ± 1.6	5.8 ± 1.8

Data are shown as mean ± standard deviation.

^*^p < 0.05 (Tukey’s honest significant difference test after one-way analysis of variance, comparison with normal group).

PUFA, polyunsaturated fatty acids.

Table 5 depicts food group consumption according to eating speed. In men, fast eaters consumed significantly more oils and coffee, and less milk, than normal-speed eaters. Slow eaters consumed significantly more soybean products, fish, green and yellow vegetables, other vegetables, mushrooms, and seaweed than the group with normal eating speed. In women, there was no difference in intake by food group according to eating speed.

**Table 5:** Consumption of dietary food groups (g/1,000 kcal) by eating speed in men and women.

	Men			Women		
Eating speed	Fast	Normal	Slow	Fast	Normal	Slow
	*n* = 863	*n* = 2,698	*n* = 314	*n* = 626	*n* = 1,873	*n* = 174
Rice	221.8 ± 70.0	225.7 ± 68.4	222.0 ± 67.3	174.5 ± 62.9	177.1 ± 59.5	174.4 ± 57.3
Bread	13.2 ± 20.7	13.0 ± 21.1	14.0 ± 20.5	19.2 ± 20.8	19.7 ± 20.9	18.6 ± 19.9
Noodles	38.5 ± 35.4	36.9 ± 32.9	35.7 ± 34.6	40.5 ± 31.8	39.6 ± 29.4	37.4 ± 26.2
Potatoes	7.0 ± 6.8	7.3 ± 7.0	8.2 ± 7.6	11.6 ± 8.8	11.9 ± 9.3	12.4 ± 10.5
Confectionary	7.9 ± 10.0	7.3 ± 8.1	7.8 ± 11.2	13.3 ± 13.3	12.2 ± 10.5	12.5 ± 11.8
Oils	7.1 ± 5.2^*^	6.6 ± 4.5	7.0 ± 5.5	10.4 ± 6.2	10.5 ± 5.9	11.3 ± 6.2
Soybean products	40.3 ± 23.6	41.2 ± 23.6	46.0 ± 26.6^*^	48.3 ± 26.3	50.0 ± 26.6	50.1 ± 24.2
Fish	28.6 ± 19.0	29.6 ± 19.2	33.6 ± 22.9^*^	35.0 ± 19.3	35.7 ± 18.0	35.9 ± 20.6
Meat	16.7 ± 10.7	16.4 ± 11.3	17.0 ± 11.0	24.4 ± 14.4	24.2 ± 12.5	24.1 ± 14.5
Eggs	8.5 ± 8.0	8.4 ± 8.4	9.1 ± 9.6	11.1 ± 8.2	11.8 ± 8.6	12.3 ± 9.5
Milk	50.7 ± 51.7^*^	56.2 ± 54.6	63.5 ± 55.2	81.7 ± 73.0	87.7 ± 67.2	99.4 ± 71.4
Green and yellow vegetables	32.8 ± 26.1	33.2 ± 27.4	38.3 ± 31.6^*^	54.8 ± 36.6	56.8 ± 37.8	60.1 ± 40.1
Other vegetables	29.4 ± 22.3	29.1 ± 21.5	32.7 ± 25.4^*^	50.4 ± 32.3	51.8 ± 31.1	53.9 ± 35.0
Fruits	21.4 ± 23.1	22.3 ± 23.0	24.1 ± 29.0	40.6 ± 37.7	42.1 ± 38.0	45.7 ± 38.1
Mushrooms	3.1 ± 3.5	3.1 ± 3.3	3.6 ± 4.1^*^	6.5 ± 5.6	6.8 ± 5.8	6.9 ± 5.9
Seaweed	0.8 ± 0.9	0.7 ± 0.9	0.9 ± 1.1^*^	1.1 ± 1.2	1.2 ± 1.2	1.1 ± 0.9
Coffee	58.7 ± 58.3^*^	50.7 ± 52.8	54.9 ± 55.4	98.1 ± 72.4	95.2 ± 68.3	83.8 ± 71.4
Green tea	114.9 ± 121.0	117.5 ± 117.4	115.9 ± 124.9	161.0 ± 146.9	170.3 ± 143.2	167.6 ± 147.2
Alcoholic beverages	135.8 ± 124.8	127.8 ± 117.0	122.9 ± 119.3	54.7 ± 84.8	48.2 ± 76.8	50.6 ± 85.8

Data are shown as mean ± standard deviation.

^*^p < 0.05 (Tukey’s honest significant difference test after one-way analysis of variance, comparison with normal group).

Table 6 shows ORs and 95% confidence intervals for the association between eating speed and visceral fat accumulation. In men, the OR for WC obesity was significantly lower in all models among slow eaters than in the group with normal eating speed. In women, there was no significant association between eating speed and WC obesity.

**Table 6:** Multivariable analysis of visceral fat accumulation by eating speed.

	Men			Women		
Eating speed	Fast	Normal	Slow	Fast	Normal	Slow
	*n* = 863	*n* = 2,698	*n* = 314	*n* = 626	*n* = 1,873	*n* = 174
Model 1	1.17	1	0.65	1.13	1	0.85
	(0.98, 1.39)	(reference)	(0.49, 0.88)^*^	(0.73, 1.76)	(reference)	(0.38, 1.88)
						
Model 2	1.15	1	0.64	1.11	1	0.83
	(0.97, 1.37)	(reference)	(0.47, 0.86)^*^	(0.71, 1.74)	(reference)	(0.37, 1.85)
						
Model 3	1.12	1	0.61	1.16	1	0.82
	(0.94, 1.34)	(reference)	(0.46, 0.83)^*^	(0.74, 1.81)	(reference)	(0.37, 1.84)

Logistic regression analysis. Data are odds ratios (95% confidence intervals). ^*^p < 0.05.

Model 1: Adjusted for age.

Model 2: Adjusted for age, dietary energy intake (kcal), dietary protein intake (%energy), dietary fat intake (%energy), dietary alcohol intake (%energy), dietary total fiber intake (g/1,000 kcal), breakfast eating habits, and daytime and nighttime snack eating habits.

Model 3: Adjusted for age, dietary energy intake (kcal), dietary protein intake (%energy), dietary fat intake (%energy), dietary alcohol intake (%energy), dietary total fiber intake (g/1,000 kcal), breakfast eating habits, daytime and nighttime snack eating habits, smoking habits, and metabolic equivalents.

## 4 Discussion

The purpose of the present study was to determine the relationship between eating speed and WC obesity in middle-aged men and women who are considered to be of normal weight according to BMI. Our findings showed that eating slowly was associated with the prevention of WC obesity among male participants in our cohort.

We used self-reported eating speed in this study, but the findings closely agree with those generated from a study among young women in which the rates of exact and adjunct agreement for eating speed measures were 46 and 47%, respectively, indicating high levels of agreement between self-reported and friend-reported eating speed (9). A Dutch study examining the association between self-reported eating speed and measured eating time also showed that measured eating times were substantially faster in the group with higher self-reported eating speeds (12). This suggests that although self-reported eating speed is based on subjective self-evaluation, it correlates well with evaluation by others and measured eating times. Therefore, the self-reported eating speeds in the present study can be considered reliable.

According to the results of the 2015 Japan National Health and Nutrition Survey, which used a cutoff value of ≥85 cm for WC in men and ≥ 90 cm for WC in women to determine visceral fat accumulation, 56.8% of men and 19.8% of women had visceral fat accumulation. Of these, 27.0% of men and 5.8% of women with a BMI of <25 kg/m^2^ had visceral fat accumulation. Using the same criteria, we found that 25.7% of men and 4.3% of women had WC obesity, which is similar to nationwide estimates for Japan. One factor associated with visceral fat accumulation is female hormones (17), but lifestyle habits such as exercise, smoking, and eating (28–31), as well as eating speed, also have an effect. Previous studies have also shown that lifestyle habits such as smoking and alcohol consumption differ between men and women (5–8, 18). In this study, we examined differences in lifestyle habits between men and women. We found that men were more likely than women to miss breakfast and smoke; to consume more rice and alcoholic beverages; to consume less meat, fish, soybean products, and vegetables as side dishes; and to bias their energy sources toward carbohydrates and alcohol. A similar trend was reported in the results of the Japan National Health and Nutrition Examination Survey, which showed that women in Japan have healthier diets with a greater variety of foods than men (18). However, we also found that women who ate quickly were more likely to eat a snack every day. As an index of physical activity, METs were higher in men and physical activity was more vigorous than in women; the fact that more men had accumulated visceral fat suggests that eating habits have a significant effect on visceral fat accumulation.

Another characteristic of obese individuals is under-reporting of dietary intake. In this study, energy intake reporting was evaluated using the Goldberg and Black method, and although there was a difference in the proportion of over-reporters between men and women, there was no difference in the proportion of over-reporters who correctly reported their energy intake. Therefore, the association between misreporting of energy intake and visceral fat accumulation was considered to be low.

Because lifestyle habits differ between men and women, the relationship between eating fast and visceral fat accumulation was examined separately for men and women. The results showed that male fast eaters were more likely to miss breakfast, and had lower percentages of not snacking and physical activity. Male fast eaters consumed significantly lower amounts of dairy products and more coffee and oils. It is possible that male fast eaters consumed snacks more frequently but in smaller amounts. It is also possible that they considered coffee a snack. As there was no difference in snack intake by food group between female slow, fast, and normal eaters, it is possible that women snacked more frequently but consumed less per meal. Smoking habits and misreporting of energy intake also did not differ according to eating speed for either sex. In women, there was no difference according to the presence or absence of menopause and eating speed, and little association between smoking, misreporting, and menopause.

Regarding nutrient intake, we found no difference in the consumption of total dietary energy, carbohydrate energy ratio, and alcohol energy ratio according to eating speed. However, men in the slow-eating group had significantly higher intakes of dietary protein; fat energy; and total, soluble, and insoluble dietary fiber than men with a normal eating speed. Dietary fiber reduces gastric emptying and slows energy and nutrient absorption, leading to lower postprandial glucose and lipid levels (32, 33). Sources of dietary fiber include plant foods such as vegetables, fruits, mushrooms, and seaweed. Epidemiological studies have shown that high dietary fiber intake is associated with a lower risk of obesity and visceral fat accumulation (34, 35). Short-chain fatty acids are produced by fermentation of intestinal bacteria using soluble dietary fiber as a substrate. Prospective cohort studies and randomized controlled trials have shown that this short-chain fatty acid stimulation can improve metabolic parameters and reduce weight and body fat mass gain in obesity (34–37). Men with slow eating speeds had greater intake of soybean products than those with normal eating speeds. Soybean products is a source of isoflavones, and has properties that resemble female hormones. Korean studies have suggested that more habitual consumption of soy protein, isoflavones, and soybean-based foods lowers the risk of female abdominal obesity (38, 39). In this study, only men showed a significant difference in soybean products intake owing to differences in eating speed. As in previous studies, this suggests that greater soybean products intake may be associated with a lower risk of WC obesity. Regarding fat intake, slow eaters had significantly higher intakes of PUFA and omega-3 PUFA than normal-speed eaters. Omega-3 PUFA are abundant in fish, and slower eaters in this study had greater intake of fish than those with normal eating speeds. Omega-3 PUFA increase energy metabolism, increase the activity and gene expression of fatty acid oxidation-related enzymes, and have lipolytic effects in adipocytes (40). It has been shown that WCs are smaller than the reference values for metabolic syndrome in individuals with a dietary pattern that includes a high intake of vegetables and fruits, as well as fish (30, 31). Therefore, the accumulation of visceral fat is not solely owing to the intake of fish and omega-3 fatty acids but may have a synergistic effect on the combination of other foods. Our study also showed that in slow eaters, following a healthy diet was effective in reducing obesity, suggesting that a combination of diet and nutritional intake may help prevent WC obesity.

Even after adjusting for the intake of these nutrients and METs, the slow-eating group of men had a significantly lower risk of WC obesity than the group with normal eating speed. The slow-eating group ate a high-fiber diet that included high-fiber vegetables, fruits, mushrooms, and seaweed, which are foods that require a lot of chewing before they can be swallowed (41). It has been reported that a higher number of chews is associated with greater diet-induced thermogenesis and greater energy expenditure. Even when eating the same food, slow eaters chew more before swallowing a bite and chew more in total before finishing their meal (42, 43). Therefore, the reason why slow eating showed a negative correlation with the risk of WC obesity in this study may be because slow eaters not only choose foods that take longer to chew but they also spend more time chewing before swallowing, which leads to greater energy expenditure and helps to reduce visceral fat accumulation. This is probably because these people choose foods that not only take longer to chew but also take longer to swallow. Therefore, it is considered that a combination of slow eating and increasing the number of chews suppress WC obesity.

In women, there was no association between eating speed and dietary content or WC obesity. Previous studies have shown that women who are fast eaters have the same low fiber intake as men, but these studies have included both obese and thin people (41). However, we found that women’s fiber intake did not differ by eating speed, and that women also consumed a greater variety of foods, had healthier eating habits with higher fiber intake, and consumed less alcohol than men. Regardless of eating speed, the fact that men have unhealthier eating habits than women may itself contribute to the risk of WC obesity. Thus, the reason women did not show an association between eating speed and WC obesity may be that the effect of eating habits, which vary widely between men and women, was stronger than that of eating speed. Moreover, it is possible that no association was found between fast eating and diet or WC obesity in women because our study was limited to participants with normal BMI.

The slow-eating group of men included significantly fewer participants with a WC of ≥85 cm than the normal group, but there was no significant association in women. Previous studies on eating speed and obesity showed that faster eating speeds are associated with a greater proportion of both men and women judged to be obese, whether according to WC, BMI, or body fat rate (9–16). In this study, we used WC as a measure of obesity, and found an association between eating speed and WC obesity only in men. This may be because previous studies have included individuals with a BMI of ≥25 kg/m^2^, whereas the present study included individuals with a BMI between 18.5 and 25.0 kg/m^2^. However, a previous study of Japanese normal body weight female college students showed that eating faster is associated with substantially higher body fat percentages (13). We used a cutoff value of ≥85 cm for men and ≥ 90 cm for women based on the metabolic syndrome criteria for Japanese individuals (4). However, some Japanese reports suggest that the WC cutoff value for metabolic syndrome is 85 cm for Japanese men and 80 cm for women, based on visceral fat volume or visceral fat area (44, 45). Thus, the different associations between eating speed and WC obesity in men and women may be owing to the WC criteria used.

There are several study limitations. First, because this was a cross-sectional study, we cannot prove that eating speed affects WC obesity. Second, because the cohort participants in this study were residents of Yamagata, a rural area of Japan, it is unknown whether the results would be similar for people living in another region. Third, because an FFQ was used to assess dietary intake, it was not possible to identify food intake status not captured in the FFQ. Therefore, the present results should be interpreted carefully.

In conclusion, the findings of this study suggest that eating slowly is associated with healthy dietary habits and can prevent the accumulation of visceral fat in men with a BMI between 18.5 and 25.0 kg/m^2^, but not in women, suggesting a sex difference. It is possible that women have healthier eating habits and consume a greater variety of foods than men, regardless of eating speed, which may have contributed to the lack of association between eating speed and visceral fat accumulation.

## Data availability statement

The data analyzed in this study is subject to the following licenses/restrictions: The dataset of the current study was not publicly available for ethical reasons. However, it can be accessed by contacting the corresponding author upon reasonable request. Requests to access these datasets should be directed to YY, y-yagu@e.yamagata-u.ac.jp.

## Ethics statement

The studies involving humans were approved by the Ethics committee of Yamagata University School of Medicine. The studies were conducted in accordance with the local legislation and institutional requirements. The participants provided their written informed consent to participate in this study.

## Author contributions

YY: Conceptualization, Formal analysis, Methodology, Writing – original draft, Writing – review & editing. TsK: Investigation, Project administration, Writing – review & editing. NI: Methodology, Writing – review & editing. CG: Methodology, Writing – review & editing. YU: Investigation, Project administration, Writing – review & editing. TaK: Investigation, Project administration, Writing – review & editing.

## References

[1] Czernichow S Kengne AP Stamatakis E Hamer M Batty GD. Body mass index, waist circumference and waist-hip ratio: which is the better discriminator of cardiovascular disease mortality risk? Evidence from an individual-participant meta-analysis of 82,864 participants from nine cohort studies. Obes Rev. (2011) 12:680–7. doi: 10.1111/j.1467-789X.2011.00879.x, PMID: 21521449 PMC4170776

[2] Jayedi A Soltani S Motlagh SZ Emadi A Shahinfar H Moosavi H. Anthropometric and adiposity indicators and risk of type 2 diabetes: systematic review and dose-response meta-analysis of cohort studies. BMJ. (2022) 376:e067516. doi: 10.1136/bmj-2021-067516 35042741 PMC8764578

[3] Xiang M Hu H Imai T Nishihara A Sasaki N Ogasawara T. Association between anthropometric indices of obesity and risk of cardiovascular disease in Japanese men. J Occup Health. (2020) 62:e12098. doi: 10.1002/1348-9585.12098 31750612 PMC6970391

[4] The Examination Committee of Criteria for ‘Obesity Disease’ in Japan. Japan Society for the Study of obesity. New criteria for ‘obesity disease’ in Japan. Circ J. (2002) 66:987–92. doi: 10.1253/circj.66.987, PMID: 12419927

[5] Sahakyan KR Somers VK Rodriguez-Escudero JP Hodge DO Carter RE Sochor O. Normal-weight central obesity: implications for total and cardiovascular mortality. Ann Intern Med. (2015) 163:827–35. doi: 10.7326/M14-2525, PMID: 26551006 PMC4995595

[6] Shirasawa T Ochiai H Yoshimoto T Nagahama S Kobayashi M Ohtsu I. Associations between normal weight central obesity and cardiovascular disease risk factors in Japanese middle-aged adults: a cross-sectional study. J Health Popul Nutr. (2019) 38:46. doi: 10.1186/s41043-019-0201-5, PMID: 31849344 PMC6918653

[7] Shirasawa T Ochiai H Yoshimoto T Nagahama S Watanabe A Yoshida R. Cross-sectional study of associations between normal body weight with central obesity and hyperuricemia in Japan. BMC Endocr Disord. (2020) 20:2. doi: 10.1186/s12902-019-0481-1, PMID: 31906920 PMC6945764

[8] Tatsumi Y Nakao YM Masuda I Higashiyama A Takegami M Nishimura K. Risk for metabolic diseases in normal weight individuals with visceral fat accumulation: a cross-sectional study in Japan. BMJ Open. (2017) 7:e013831. doi: 10.1136/bmjopen-2016-013831, PMID: 28093438 PMC5253636

[9] Sasaki S Katagiri A Tsuji T Shimoda T Amano K. Self-reported rate of eating correlates with body mass index in 18-y-old Japanese women. Int J Obes Relat Metab Disord. (2003) 27:1405–10. doi: 10.1038/sj.ijo.0802425, PMID: 14574353

[10] Sonoda C Fukuda H Kitamura M Hayashida H Kawashita Y Furugen R. Associations among obesity, eating speed, and oral health. Obes Facts. (2018) 11:165–75. doi: 10.1159/000488533, PMID: 29669358 PMC5981670

[11] Tanihara S Imatoh T Miyazaki M Babazono A Momose Y Baba M. Retrospective longitudinal study on the relationship between 8-year weight change and current eating speed. Appetite. (2011) 57:179–83. doi: 10.1016/j.appet.2011.04.017, PMID: 21565235

[12] van den Boer JHW Kranendonk J van de Wiel A Feskens EJM Geelen A Mars M. Self-reported eating rate is associated with weight status in a Dutch population: a validation study and a cross-sectional study. Int J Behav Nutr Phys Act. (2017) 14:121. doi: 10.1186/s12966-017-0580-1, PMID: 28886719 PMC5591506

[13] Yaguchi-Tanaka Y Kawagoshi Y Sasaki S Fukao A. Cross-sectional study of possible association between rapid eating and high body fat rates among female Japanese college students. J Nutr Sci Vitaminol (Tokyo). (2013) 59:243–9. doi: 10.3177/jnsv.59.243, PMID: 23883696

[14] Ohkuma T Hirakawa Y Nakamura U Kiyohara Y Kitazono T Ninomiya T. Association between eating rate and obesity: a systematic review and meta-analysis. Int J Obes. (2015) 39:1589–96. doi: 10.1038/ijo.2015.96, PMID: 26100137

[15] Iwasaki T Hirose A Azuma T Watanabe K Deguchi F Obora A. Self-reported behavior of eating quickly is correlated with visceral fat area in Japanese non-obese adults. Asia Pac J Clin Nutr. (2019) 28:92–8. doi: 10.6133/apjcn.201903_28(1).0013, PMID: 30896419

[16] Wuren, Endoh K. Kuriki K, the Shizuoka-Sakuragaoka J-MICC Study group. Eating rate as risk for body mass index and waist circumference obesity with appropriate confounding factors: a cross-sectional analysis of the Shizuoka-Sakuragaoka J-MICC study. Asia Pac J Clin Nutr. (2019) 28:79–91. doi: 10.6133/apjcn.201903_28(1).0012, PMID: 30896418

[17] Frank AP de Souza SR Palmer BF Clegg DJ. Determinants of body fat distribution in humans may provide insight about obesity-related health risks. J Lipid Res. (2019) 60:1710–9. doi: 10.1194/jlr.R086975, PMID: 30097511 PMC6795075

[18] Murakami K Livingstone MBE Okubo H Sasaki S. Energy density of the diets of Japanese adults in relation to food and nutrient intake and general and abdominal obesity: a cross-sectional analysis from the 2012 National Health and nutritional survey. Japan Br J Nutr. (2017) 117:161–9. doi: 10.1017/S0007114516004451, PMID: 28112076

[19] Yamagata University Genomic Cohort Consortium, Narimatsu H. Constructing a contemporary gene-environmental cohort: study design of the Yamagata molecular epidemiological cohort study. J Hum Genet. (2013) 58:54–6. doi: 10.1038/jhg.2012.128 23171998

[20] Tokudome S Goto C Imaeda N Tokudome Y Ikeda M Maki S. Development of a data-based short food frequency questionnaire for assessing nutrient intake by middle-aged Japanese. Asian Pac J Cancer Prev. (2004) 5:40–3. PMID: 15075003

[21] Tokudome Y Goto C Imaeda N Hasegawa T Kato R Hirose K. Relative validity of a short food frequency questionnaire for assessing nutrient intake versus three-day weighed diet records in middle-aged Japanese. J Epidemiol. (2005) 15:135–45. doi: 10.2188/jea.15.135 16141632 PMC7851066

[22] Imaeda N Goto C Tokudome Y Hirose K Tajima K Tokudome S. Reproducibility of a short food frequency questionnaire for Japanese general population. J Epidemiol. (2007) 17:100–7. doi: 10.2188/jea.17.100, PMID: 17545697 PMC7058456

[23] Imaeda N Goto C Sasakabe T Mikami H Oze I Hosono A. Reproducibility and validity of food group intake in a short food frequency questionnaire for the middle-aged Japanese population. Environ Health Prev Med. (2021) 26:28. doi: 10.1186/s12199-021-00951-3, PMID: 33653279 PMC7923820

[24] Black AE. Critical evaluation of energy intake using the Goldberg cut-off for energy intake: basal metabolic rate. A practical guide to its calculation, use and limitations. Int J Obesity. (2000) 24:1119–30. doi: 10.1038/sj.ijo.0801376, PMID: 11033980

[25] Miyake R Tanaka S Ohkawara K Ishikawa-Takata K Hikihara Y Taguri E. Validity of predictive equations for basal metabolic rate in Japanese adults. J Nutr Sci Vitaminol. (2011) 57:224–32. doi: 10.3177/jnsv.57.224, PMID: 21908945

[26] Kikuchi H Inoue S Odagiri Y Ihira H Inoue M Sawada N. Intensity-specific validity and reliability of the Japan public health center-based prospective study-physical activity questionnaire. Prev Med Rep. (2020) 20:101169. doi: 10.1016/j.pmedr.2020.101169, PMID: 32884896 PMC7452103

[27] Craig CL Marshall AL Sjöström M Bauman AE Booth ML Ainsworth BE. International physical activity questionnaire: 12-country reliability and validity. Med Sci Sports Exerc. (2003) 35:1381–95. doi: 10.1249/01.MSS.0000078924.61453.FB, PMID: 12900694

[28] Uemura H Katsuura-Kamano S Iwasaki Y Arisawa K Hishida A Okada R. Independent relationships of daily life and leisure-time exercise with metabolic syndrome and its traits in the general Japanese population. Endocrine. (2019) 64:552–63. doi: 10.1007/s12020-019-01926-9, PMID: 31011988

[29] Kim JH Shim KW Yoon YS Lee SY Kim SS Oh SW. Cigarette smoking increases abdominal and visceral obesity but not overall fatness: an observational study. PLoS One. (2012) 7:e458158. doi: 10.1371/journal.pone.0045815, PMID: 23029258 PMC3454366

[30] Kanerva N Kaartinen NE Schwab U Lahti-Koski M Männistö S. Adherence to the Baltic Sea diet consumed in the Nordic countries is associated with lower abdominal obesity. Br J Nutr. (2013) 109:520–8. doi: 10.1017/S0007114512001262 22575060

[31] Bahari T Uemura H Katsuura-Kamano S Yamaguchi M Nakamoto M Miki K. Nutrient-derived dietary patterns and their association with metabolic syndrome in a Japanese population. J Epidemiol. (2018) 28:194–201. doi: 10.2188/jea.JE20170010, PMID: 29151477 PMC5865010

[32] Shimizu H Ohue-Kitano R Kimura I. Regulation of host energy metabolism by gut microbiota-derived short-chain fatty acids. Glycative Stress Res. (2019) 6:181–91. doi: 10.24659/gsr.6.3_181

[33] Slavin JL. Dietary fiber and body weight. Nutrition. (2005) 21:411–8. doi: 10.1016/j.nut.2004.08.018 15797686

[34] Grooms KN Ommerborn MJ Pham DQ Djousse L Clark CR. Dietary fiber intake and cardiometabolic risks among US adults, NHANES 1999-2010. Am J Med. (2013) 126:1059–4. doi: 10.1016/j.amjmed.2013.07.023 24135514 PMC3865784

[35] Zamanillo-Campos R Chaplin A Romaguera D Abete I Salas-Salvadó J Martín V. Longitudinal association of dietary carbohydrate quality with visceral fat deposition and other adiposity indicators. Clin Nutr. (2022) 41:2264–74. doi: 10.1016/j.clnu.2022.08.008, PMID: 36084360 PMC9529821

[36] Hairston KG Vitolins MZ Norris JM Anderson AM Hanley AJ Wagenknecht LE. Lifestyle factors and 5-year abdominal fat accumulation in a minority cohort: the IRAS family study. Obesity (Silver Spring). (2012) 20:421–7. doi: 10.1038/oby.2011.171, PMID: 21681224 PMC3856431

[37] Huwiler VV Schönenberger KA Segesser von Brunegg A Reber E Mühlebach S Stanga Z. Prolonged isolated soluble dietary fibre supplementation in overweight and obese patients: a systematic review with meta-analysis of randomised controlled trials. Nutrients. (2022) 14:2627. doi: 10.3390/nu14132627, PMID: 35807808 PMC9268533

[38] Jun SH Shin WK Kim Y. Association of soybean food intake and cardiometabolic syndrome in Korean women: Korea National Health and nutrition examination survey (2007 to 2011). Diabetes Metab J. (2020) 44:143–57. doi: 10.4093/dmj.2019.0078, PMID: 31950771 PMC7043982

[39] Woo HW Kim MK Lee YH Shin DH Shin MH Choi BY. Habitual consumption of soy protein and isoflavones and risk of metabolic syndrome in adults ≥40 years old: a prospective analysis of the Korean multi-rural communities cohort study (MRCohort). Eur J Nutr. (2019) 58:2835–50. doi: 10.1007/s00394-018-1833-8, PMID: 30264377

[40] Albracht-Schulte K Kalupahana NS Ramalingam L Wang S Rahman SM Robert-McComb J. Omega-3 fatty acids in obesity and metabolic syndrome: a mechanistic update. J Nutr Biochem. (2018) 58:1–16. doi: 10.1016/j.jnutbio.2018.02.012, PMID: 29621669 PMC7561009

[41] Murakami K Sasaki S Takahashi Y Uenishi K Yamasaki M Hayabuchi H. Hardness (difficulty of chewing) of the habitual diet in relation to body mass index and waist circumference in free-living Japanese women aged 18-22 y. Am J Clin Nutr. (2007) 86:206–13. doi: 10.1093/ajcn/86.1.206, PMID: 17616782

[42] Ekuni D Furuta M Takeuchi N Tomofuji T Morita M. Self-reports of eating quickly are related to a decreased number of chews until first swallow, total number of chews, and total duration of chewing in young people. Arch Oral Biol. (2012) 57:981–6. doi: 10.1016/j.archoralbio.2012.02.001, PMID: 22381534

[43] Hamada Y Kashima H Hayashi N. The number of chews and meal duration affect diet-induced thermogenesis and splanchnic circulation. Obesity (Silver Spring). (2014) 22:E62–9. doi: 10.1002/oby.20715, PMID: 24493207

[44] Tsukiyama H Nagai Y Matsubara F Shimizu H Iwamoto T Yamanouchi E. Proposed cut-off values of the waist circumference for metabolic syndrome based on visceral fat volume in a Japanese population. J Diabetes Investig. (2016) 7:587–93. doi: 10.1111/jdi.12454, PMID: 27181599 PMC4931210

[45] Hara K Matsushita Y Horikoshi M Yoshiike N Yokoyama T Tanaka H. A proposal for the cutoff point of waist circumference for the diagnosis of metabolic syndrome in the Japanese population. Diabetes Care. (2006) 29:1123–4. doi: 10.2337/diacare.2951123, PMID: 16644651

